# Differentiating Interpreting Types: Connecting Complex Networks to Cognitive Complexity

**DOI:** 10.3389/fpsyg.2021.590399

**Published:** 2021-09-17

**Authors:** Yumeng Lin, Duo Xu, Junying Liang

**Affiliations:** ^1^Department of Linguistics, Zhejiang University, Hangzhou, China; ^2^School of Foreign Languages and Cultures, Nanjing Normal University, Nanjing, China

**Keywords:** interpreting types, holistic features, complex networks, cognitive load, interpreting mechanisms

## Abstract

Prominent interpreting models have illustrated different processing mechanisms of simultaneous interpreting and consecutive interpreting. Although great efforts have been made, a macroscopic examination into interpreting outputs is sparse. Since complex network is a powerful and feasible tool to capture the holistic features of language, the present study adopts this novel approach to investigate different properties of syntactic dependency networks based on simultaneous interpreting and consecutive interpreting outputs. Our results show that consecutive interpreting networks demonstrate higher degrees, higher clustering coefficients, and a more important role of function words among the central vertices than simultaneous interpreting networks. These findings suggest a better connectivity, better transitivity, and a lower degree of vocabulary richness in consecutive interpreting outputs. Our research provides an integrative framework for the understanding of underlying mechanisms in diverse interpreting types.

## Introduction

Interpreting is an extremely intricate language processing task for the cognitive system (Christoffels et al., 2006; Tzou et al., 2012; Pöchhacker, 2016; Defrancq and Plevoets, 2018; Liang et al., 2019), which involves listening and comprehending of the input speech, memory storage, production of target equivalents, and sometimes alternately activating and suppressing production in two languages (Christoffels et al., 2003; Aparicio et al., 2017; Lin et al., 2018). Among different types of interpreting, simultaneous interpreting (SI) and consecutive interpreting (CI) tasks are generally supposed to be highly cognitive demanding and with sheer complexity. Characterized by the simultaneity of input comprehension and output production, SI involves temporary storage and extraction of meaning (Christoffels et al., 2003), reformulation of the earlier segments of the source message into the target language, and articulation of even earlier segments (Gerver, 1976; Padilla et al., 1995). By contrast, CI is performed in such cases where the stages of perception and production are processed serially. Interpreters prefer to finish a complete session before he “pauses for interpretation,” such as in international press conferences. Faced with the need to render speeches lasting up to a few minutes or more, interpreters may resort to note-taking to assist memorization (Gile, 2009; Pöchhacker, 2011).

Regardless of the interpreting modes, it has been substantiated in a volume of research that cognitive resources are crucial to the interpreting performances (Padilla et al., 1995; Tzou et al., 2012; Injoque-Ricle et al., 2015; Plevoets and Defrancq, 2018; Liang and Lv, 2020). Cognitive load is defined as a “multi-dimensional construct representing the load imposed on the working memory during performance of a cognitive task” (Paas and Van Merriënboer, 1994). This construct is based on models of working memory which is limited in capacity and duration (Baddeley, 1992). According to Baddeley’s (1992) model, only a limited number of discrete items can be stored and manipulated in working memory at the same time. Specifically, Miller (1956) suggested that normal individuals can only hold seven (plus or minus two) discrete units in working memory at one time and only for limited duration (Chen et al., 2016). Given that interpreting is a complex task where extremely high demands are placed on working memory, the explanatory potential of the concept of cognitive load for the interpreting process is manifest. Gile (1985) introduced the notion of cognitive load into the field of interpreting studies, aiming to explain information loss observed in professional interpreters (Pöchhacker, 2015). He argued that interpreting failures are ascribed to deficient cognitive capacity instead of insufficient linguistic or extralinguistic knowledge of interpreters (Gile, 1999, 2009). This conforms to the “Tightrope Hypothesis” which postulates that interpreters generally work close to their maximum capacity of cognitive load (saturation) when performing the interpreting tasks (Gile, 1999).

Such severe cognitive constraints can be elaborated by theoretical models of SI and CI, which highlight the critical role of cognitive capacity in each proportion of efforts in the interpreting activities. The composition and allocation of mental operations during the processing of SI are best captured by the Effort Model (Gile, 2009, 2016), in which SI can be conceptualized as a process consisting of listening and analysis effort, the short-term memory effort, the speech production effort, and a coordination effort. These efforts are largely non-automatic and concurrent and thus compete for the limited cognitive resources, suggesting that the increments of one effort are at the expense of another (Koshkin et al., 2018). By contrast, CI is processed in two phases, namely the comprehension phase and the reformulation phase (Gile, 2009, 2016). To ensure the smooth production in SI and CI, the processing capacity available must exceed the capacity required; otherwise, errors or infelicities may occur due to insufficient cognitive resources.

The above-mentioned models, albeit conceptually presented only, explain that there exist different cognitive mechanisms underlying SI and CI, indicating that the processes of SI and CI may consume different amount of cognitive resource. This stimulates discussions on whether the difference of cognitive load between SI and CI may influence interpreting production. The disentanglement of this issue can be of great significance to our understanding of both cognitive processing and language use. On the one hand, the cognitive load of different interpreting types reflected by the quantifications of interpreting outputs helps explore the coping mechanism of interpreters. On the other hand, it also illustrates how language shifts under the extreme cognitive load, and hence complements comparative studies on fully fledged common language uses (Liang et al., 2018; Lv and Liang, 2019; Jia and Liang, 2020).

The recent 10years has witnessed booming interest in the research of cognitive load in interpreting. Some of current attempts concern capturing and understanding the difficult and demanding nature of the task as well as figuring out how interpreters deal with the challenges (Chen, 2017). Seeber (2011) postulated that cognitive load in SI varies according to the micro-strategy (waiting, stalling, text chunking, and anticipation) used by interpreters between syntactically different languages, and further suggested that cognitive load during SI of syntactically asymmetrical structures increased toward the end of the sentence (Seeber and Kerzel, 2012). Quite in the same vein, Shao and Chai (2020) found a significant drop in SI performance occurred when local cognitive load reached four chunks, corroborating the notion that interpreters generally experience cognitive saturation even in relatively easy SI tasks. Bóna and Bakti (2020) examined the effect of cognitive load on temporal and disfluency patterns between CI and sight translation and found that sight translation generated more cognitive load. Additionally, a latest study demonstrated a positive correlation between cognitive load and explicitating shifts in SI (Gumul, 2021).

Indeed, one of the major challenges in applying the construct of cognitive load to research in interpreting has been the difficulty of measuring this notion (Pöchhacker, 2015). Apart from some techniques testing brain activations or pupillary response in the experiments, language complexity can be used as a potential measure to monitor cognitive load (Chen et al., 2016; Chen 2017). For instance, Plevoets and Defrancq (2016) operationalized cognitive load in interpreting in terms of linguistic features such as delivery rate and lexical density, and a high source text delivery rate and a high target text lexical density were determinants triggering significantly higher disfluencies in the interpretations. Similar measurement was also adopted in the research by Lv and Liang (2019). To systematically probe into the connection between cognitive load and interpreting, a line of quantitative research examined the interpreting outputs between SI and CI from various perspectives. As a seminal research quantifying different interpreting modes, Liang et al. (2017) calculated the dependency distance of output texts and suggested that CI bears heavier cognitive demands than SI. Later on, they found consistent evidence in the lexical features (Lv and Liang, 2019) and language sequences (Liang et al., 2019). A more recent study (Jia and Liang, 2020) probed into the lexical category realm with the activity index and found that CI outputs yield greater activity than SI outputs, indicating “a dynamic adaptive mechanism of language representations to accommodate cognitive constraints.”

Clearly, prior studies on the comparison of SI and CI cognitive processes were conducted from syntactic, lexical and language sequential perspectives, but without taking an interconnected and comprehensive feature into consideration. Since language is represented in an integrated way, the complex network (Newman, 2018), as a systematic approach, may be a possible solution to investigate the features of different interpreting types from a macroscopic view. Therefore, we employ a novel approach of complex network to pin down the characteristics of interpreting production motivated by different cognitive constraints during SI and CI.

**Figure 1:**
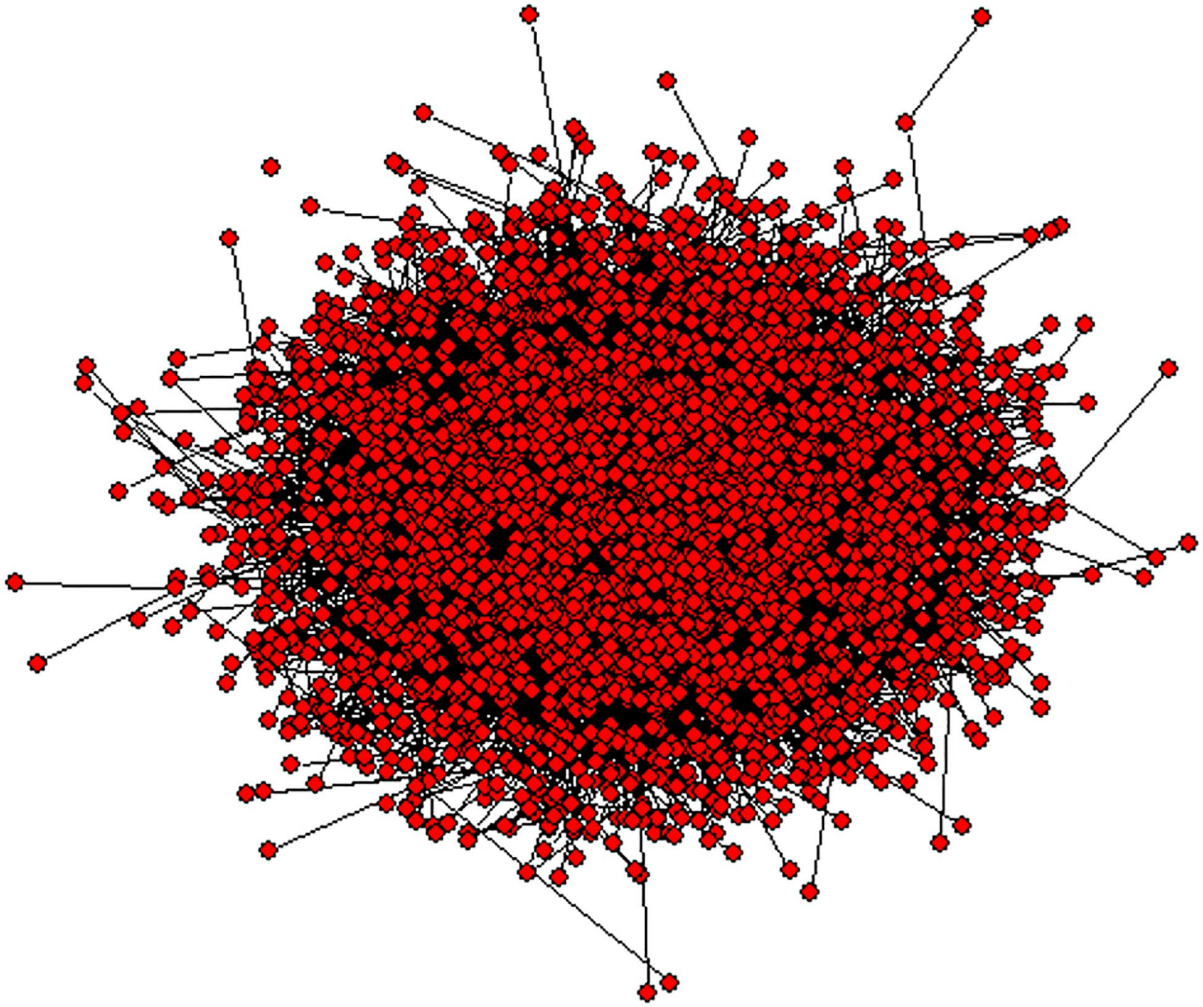
Overall view of a language network.

The rationale of complex networks as an operational approach for linguistic inquiry is from the representative theories which posit language as a system (Saussure, 2001; Hudson, 2007). Such a system is deemed as a network of relations which can be described by a number of vertices and edges. The vertices represent entities, while edges denote relationships among the vertices (Barabási, 2002; Čech et al., 2007). Linguistic networks generally exhibit scale-free structure (Liu, 2008a; Čech and Mačutek, 2009), where only a small number of vertices have extremely great combinatorial capacity, while most vertices have rather low combinatorial capacity. Among all these vertices, the well-connected vertices in the networks serve as hubs, which play an important role in the topology of a linguistic network. Displayed in Figure 1 is a panorama of dependency syntactic network based on the language data obtained from speeches on the international forums. Although there are only about 3,000 vertices in this network, it has already presented a clear picture of the complexity. Also, language sub-systems of various types generally possess the small-world structure, where there is a low degree of separation between vertices and a high level of clustering (Cong and Liu, 2014a). Such a structure can enhance the communication efficiency between the vertices and thus facilitates mental navigation (Ferrer-i-Cancho, 2005; Vitevitch, 2008). Complex networks thus provide a quantitative measure and an interdisciplinary context to explore the properties of linguistic units at the system level (Cong and Liu, 2014b).

Prior studies demonstrate the feasibility of using the complex network approach to characterize and classify human languages, including the exploration of statistical patterns of different languages, stylistic research (Ferrer-i-Cancho et al., 2004; Liu, 2008a), typological properties of languages (Liu and Xu, 2011), hierarchical structures of a language (Chen et al., 2015), the evolution of languages (Cui et al., 2017; Chen et al., 2018), and linguistic features in language learning (Jin and Liu, 2016; Jiang et al., 2019; Hao et al., 2021). It is probable that network properties can also discriminate linguistic features of different interpreting types. By utilizing this approach to interpreting, it is likely to examine the quantitative properties of interpreting outputs at the system level. In other words, both the linguistic units and their relations can be taken into consideration.

Meanwhile, given that the ultimate goal of our study is to explore whether different cognitive processing in SI and CI has impact on language production during SI and CI, it will be beneficial to adopt an approach which can effectively connect to the cognitive constructs. In this regard, the network approach is a good choice to realize our goal, since this approach has been proved to be advantageous for the representation of the cognitive system by capturing the interaction of structure and processes in the networks (Siew et al., 2019). For instance, research concerning human semantic memory conceptualizes cognitive system as networks in which concepts are connected based on their semantic similarity to account for behavioral phenomena (Collins and Loftus, 1975; Steyvers and Tenenbaum, 2005). Besides, cognitive impairment (Lerner et al., 2009), lexical retrieval (Goldstein and Vitevitch, 2017; Siew, 2018) and human navigation (Sudarshan Iyengar et al., 2012) are frequently investigated *via* the network science. The network representations serve as a mental map of the cognitive space, according to which, we can make predictions about how structural properties of such network maps influence cognitive search behavior (Siew et al., 2019). Collectively, research in this domain demonstrates the accessibility and feasibility of this measure to exploit cognitive constructs.

To examine the quantitative properties between different interpreting types at the system level and to connect these properties to cognitive constraints, here in the current research, we intend to build syntactic dependency networks. The adoption of the syntactic complex network approach has two primary considerations. On the one hand, syntax is a fundamental feature of language that concerns the organization of words and the structure of sentences. Hence, it serves as an essential foundation for understanding the processes of communication. Prior research demonstrated that dependency distance, the number of words intervening between two syntactically related words in a sentence, can reflect the cognitive constraints during varying interpreting tasks (Liang et al., 2017; Liu et al., 2017). This suggests that the syntactic feature is an applicable dimension to reflect the cognitive load. The employment of syntactic dependency networks thus conforms to the aim of our study. On the other hand, syntactic dependency networks based on syntactic relations are not related to the text content. However, the features of other types of networks such as co-occurrence networks and semantic networks can easily be affected by the content of texts. The choice of syntactic networks can exclude the influence of this factor. Given the two reasons above, the present study employs the syntactic dependency network approach.

To sum up, firstly, prior quantitative investigations into the underlying differences across different interpreting types generally focus on one particular aspect, whereas a global and systematic view into the macroscopic features between SI and CI is rather limited. The network approach is beneficial in that all the words in a text can be linked together and generate a holistic picture, instead of investigating linguistic features based on separated sentences. Secondly, the network approach proved to be very fruitful in examining linguistic features systematically and representing cognitive constructs. Thus, complex networks may facilitate our understanding of the properties of interpreting outputs under the extreme cognitive load, providing insights into the interconnection between language and cognitive sciences.

The present study therefore investigates different properties of syntactic dependency networks between SI and CI outputs, to explore whether different underlying mechanisms between SI and CI can influence interpreting production in SI and CI. The following questions will be addressed:

1. Do the syntactic networks of CI and SI outputs display the scale-free and small-world network structures?
2. What are the differences in the cognitive complexity between SI and CI processes reflected by the main properties of syntactic networks?
3. Can the characteristics of central vertices in the networks discriminate SI and CI?

## Materials and Methods

### Corpus Descriptions

We adopted the transcribed real-world interpretations and the input texts of SI and CI as the corpus of the current research. The CI sub-corpus consisted of speeches given by Chinese Premiers Wen Jiabao and Li Keqiang at the annual press conference of two sessions (the National People’s Congress and the Chinese Political Consultative Conference), where the Prime Minister met and answered the questions raised by Chinese and overseas journalists. The SI sub-corpus comprised keynote speeches delivered by Chinese government leaders such as Wen Jiabao, Li Keqiang, and Xi Jinping on the international forums including sessions of the UN General Debate, the Davos Forum, the BRICS summit, the Boao Forum for Asia, the G20 Summit, the World Economic Forum, the ASEM Summit, and China-ASEAN Business and Investment Summit during the same time span. All the corresponding interpretations were from the interpreters’ mother tongue (Mandarin Chinese) into their second language (English).

To maximize the consistency of the text content, our corpus was composed of 12 SI input texts and 12 CI input texts, together with their corresponding outputs, without synthesizing all the texts into one. Therefore, all together, we built 24 input networks and the corresponding 24 networks of SI and CI. To ensure comparability and integrality, each text was trimmed to be of similar length without separating a complete paragraph, and the alignment between input and output on the sentence level was also taken into consideration. The summary of the corpus is presented in Table 1.

**Table 1:** Summary of the selected texts in the corpus.

Interpreting types	Text types	Number	Size (tokens)
CI	IT	12	55,692
	OT	12	75,211
SI	IT	12	59,141
	OT	12	77,149
Total		48	267,193

IT, input texts; OT, output texts.

The input speeches in the CI and SI corpus are comparable in the following aspects. First, the input texts of CI and SI were all public speeches delivered on internationally high-level conferences from 2007 to 2018, with equivalent topic areas in political and economic fields. Thus, the source speeches were homogeneous in formality, delivery rate, language register, topic area, and time span. Second, the results of type–token ratio (TTR), an indicator of lexical diversity, showed that no significant difference was observed between CI and SI input speeches (*t*=−0.775, *p*=0.447), ensuring the comparability of the input speeches in terms of lexical diversity. Third, as for the syntactic complexity, a previous study of Liang et al. (2017) using an identical corpus computed the mean dependency distance (MDD) of the input speeches and demonstrated that SI and CI input speeches are homogeneous in terms of syntactic complexity. Fourth, regarding the speakers and interpreters, all the speeches were addressed by the Chinese government heads and interpreted by top-level professional interpreters from the Department of Translation and Interpretation of China’s Ministry of Foreign Affairs, and both the CI and SI interpreters were seated at a table or in an interpreting booth during interpreting, ensuring the consistency of the speaking and interpreting style.

### The Construction of Networks

**Figure 2:**
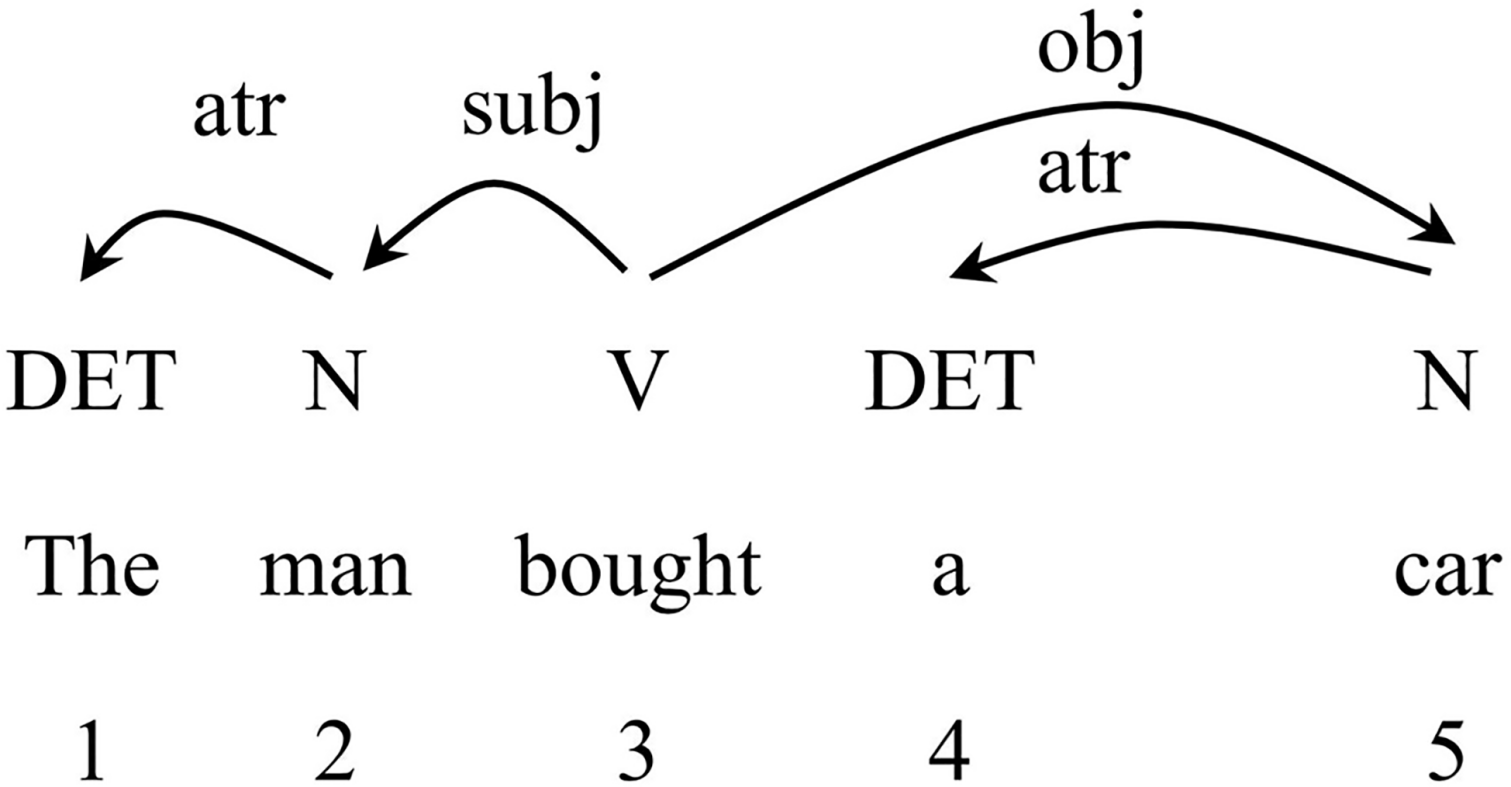
Dependency structure of sample sentence “*The man bought a car.*”

The focus of our study is to build the syntactic dependency networks of SI and CI interpreting texts, in which vertices represent the word forms, and edges are syntactic dependencies between them (Liu, 2008a). A syntactic dependency network is usually converted from a dependency-annotated tree bank which is constructed with dependency grammar (Cong and Liu, 2014a). Dependency grammar is a syntactic analysis approach which concerns asymmetric pairwise relations with one of the two-word units as Governor and the other as Dependent (Mel’čuk, 1988). Each pair of relations is indicated by a directed arc pointing from Governor to Dependent with a label of dependency type on the top of the sentence. Given that dependency analysis involves binary relation between two linguistic units (Nivre, 2006; Hudson, 2007), it is more suitable to be employed in the network analysis. From the perspective of the complex network, the dependent and the governor are vertices, and the links of dependency are edges (Liu, 2008a). The analysis of the sentence “*The man bought a car*” using dependency grammar is illustrated as in Figure 2.

Treebanks are syntactically annotated corpus based on dependency analysis (Abeillé, 2003), from which the syntactic dependency networks can be constructed. Therefore, to start with, we built a dependency-annotated treebank of authentic speeches based on the input texts and the interpreted texts of SI and CI. The syntactic treebanks were automatically built by Stanford Parser 3.6.0 (Marneffe and Manning, 2008), and the annotation accuracy was manually checked. Then, two columns of words which represent the governors and the dependents were extracted from the treebanks. Hence, the dependency relation of the example sentence extracted from its treebank is displayed in Table 2.

**Table 2:** Dependency relations of the sample sentence extracted from the treebank.

	Dependent	Governor
1	The	man
2	man	bought
3	bought	
4	a	car
5	car	bought

**Figure 3:**
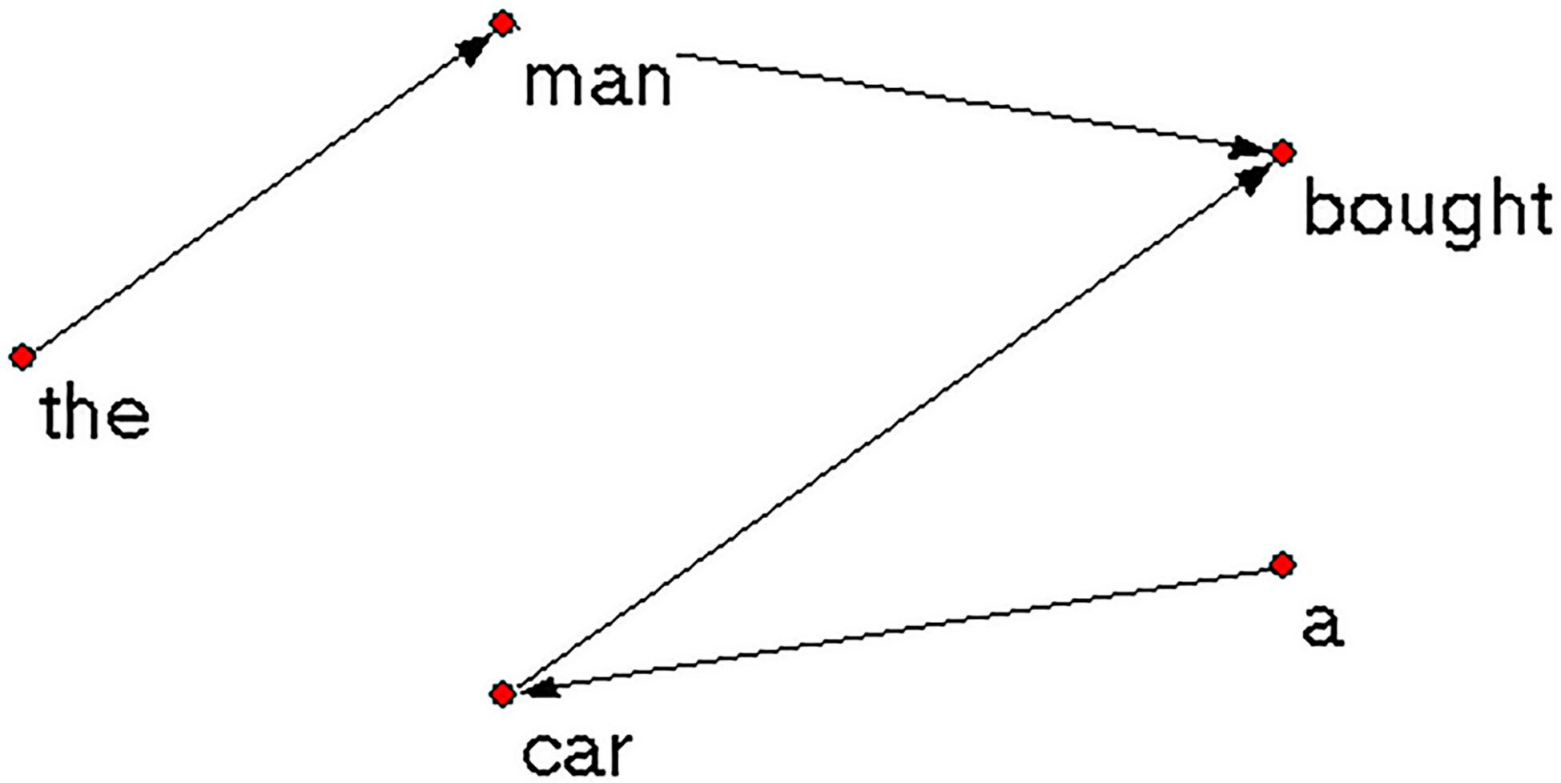
An example of syntactic dependency network.

In this way, a list of binary relations of all the words in each text were generated in the EXCEL which can be easily converted into the net. File format by Createpajek^1^ (a network converting software). These files were then put into Pajek^2^ (version 5.06) to construct syntactic dependency networks. The network of the sample sentence built by Pajek is shown in Figure 3. The parameters of each syntactic dependency network were calculated, which would be introduced in the following section.

### Network Parameters

A multitude of quantitative measures were adopted to investigate the characterization of complex networks (Newman, 2010; Estrada, 2011). The present study focuses on some commonly used measures of a linguistic network involving degree, average degree, clustering coefficient, average path length, diameter, density, centrality, and small-world and scale-free structures.

In a linguistic network, a vertex’s degree refers to the number of edges which connect to it, representing the connectivity of a linguistic unit in the language sub-system. In a syntactic dependency network, degree denotes the range of a word’s possible dependency connections with other words (Chen et al., 2011). It is a measure for the corresponding word’s combinatorial capacity to form syntactic dependency relations.

The average degree (⟨k⟩) is the mean of degrees of all its vertices, frequently used to estimate any given linguistic unit’s connectivity (number of edges) in the sub-system:

$$k=\frac{1}{N}\underset{i}{\sum }k_{i}$$ (1)

In this formula, N denotes the number of vertices, and *k_i_* represents the number of edges of the vertex *i* in the network.

The clustering coefficient C is defined as a measure of the degree to which the vertices in a network tend to cluster together, or of the patterns of clusters. It reflects the proportion of a node’s neighbors that are themselves neighbors, or the degree to which a cluster’s connectivity is perfect (Cong and Liu, 2014b). The clustering coefficient *C_i_* of a vertex *i* is formulated as:

$$C_{i}=\frac{2E_{i}}{k_{i}\left(k_{i}-1\right)}$$ (2)

where *E_i_* is the number of edges among the vertices in the nearest neighbors of vertex *i* (Liu, 2008a) and *k_i_* is the degree of vertex *i* or the number of neighbors that the vertex *i* has (Watts and Strogatz, 1998). The clustering coefficient of the network is defined by the average of *Ci* over all the vertices in the network:

$$C=\frac{1}{N}\overset{n}{\underset{i=1}{\sum }}C_{i}$$ (3)

The average path length L is the average distance of the shortest path length over all possible pairs of vertices, which represents the degree of separation between a pair of linguistic units:

$$L=\frac{2}{N\left(N-1\right)}\underset{i>j}{\sum }d_{ij}$$ (4)

where N is the number of vertices in the network and *d_ij_* is the distance of the minimal path between vertex *i* and vertex *j*.

Diameter *D* is the maximal shortest path length in a network.

Density (ρ) is defined as “the ratio of the actual number of edges in the network to the maximal possible number of edges (Cong and Liu, 2014a).”

The centrality of a node reflects its importance and authority in the complex network. It is usually defined by different measures, such as degree centrality, betweenness centrality and closeness centrality (Jin and Liu, 2016). Degree centrality quantifies the number of edges which are directly linked to the vertices. Betweenness centrality is calculated by the number of the shortest distance paths passing through a vertex *v*, which is defined as:

$$C_{B}\left(v\right)=\underset{i\neq j}{\sum }\frac{G_{v}\left(i,j\right)}{G\left(i,j\right)}$$ (5)

where *G_v_* (*i*, *j*) is the number of shortest pathways between *i* and *j* running through *v* and *G*(*i*, *j*)=∑*_v_G_v_*(*i*, *j*) (Ferrer-i-Cancho et al., 2004).

Based on the above-mentioned parameters, the scale-free and small-world properties of a network can be evaluated. The scale-free property is examined by degree distribution which is presented by a distribution function *P*(*k*), representing the probability that a randomly chosen node will have degree *k*.

Generally, the real-world networks have the characteristics of the scale-free feature, that is, the degree distribution of a linguistic complex network follows the power-law formula (Barabási and Albert, 1999; Caldarelli, 2007):

$$P\left(k\right)∼k^{-\gamma }$$ (6)

The scale-free feature indicates that only a small number of vertices have extremely high degrees whereas most vertices have rather low degrees (Cong and Liu, 2014a).

The small-world property is evaluated by the relations between the average path length L and the clustering coefficient C of an actual network and those of its corresponding random network which has the same number of vertices and edges as the actual one. If a network satisfies the condition that *L*~*L_rand_* and C≫*C_rand_*, it is a small-world with the feature of a low degree of separation between vertices and high level of clustering (Watts and Strogatz, 1998; Cong and Liu, 2014a). Humphries and Gurney (2008) also define a precise measure of small-worldness which is denoted as:

$$S=\frac{C/C_{rand}}{L/L_{rand}}$$ (7)

Small-world networks often have S≫1 (Humphries and Gurney, 2008; Rubinov and Sporns, 2010).

## Results

We first checked the equivalence of the text size of inputs by an independent-samples *t*-test to ensure the comparability of two sets of networks. Then, one-way ANCOVAs were performed to examine the differences of the network parameters between SI and CI by excluding the interference from the input values of the parameters. The values of each input network parameter were used as a covariate, and the interpreting mode was utilized as an independent variable. The values of each output network parameter were adopted as a dependent variable. Furthermore, to investigate the features of the hubs in the two interpreting modes, the proportion of function words in the top-ranking values of centrality was calculated and the differences were evaluated by a Mann–Whitney U test.

The sizes of CI and SI input texts conformed to the normal distribution, and no outlier was detected. The independent-samples *t* test indicated no significant difference in the sizes of SI and CI input texts, *t* (22)=−1.954, *p*>0.05.

### Small-World and Scale-Free Properties of SI and CI Networks

**Figure 4:**
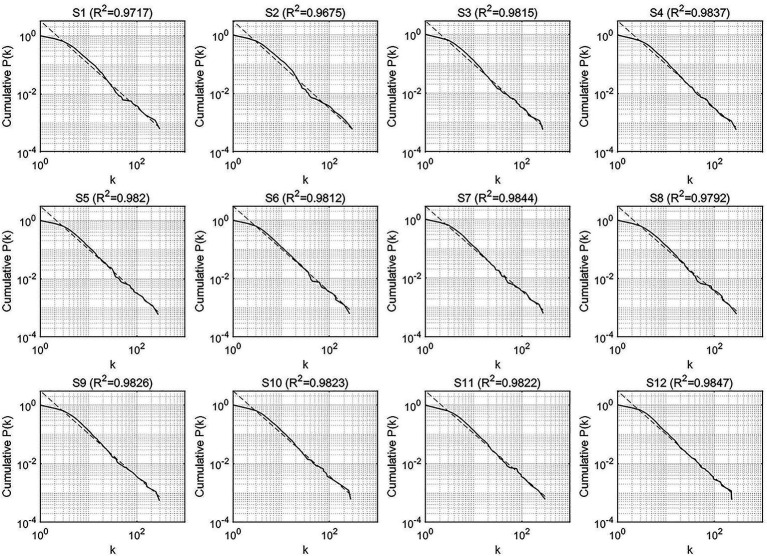
The cumulative degree distributions of the syntactic dependency networks of CI outputs.

**Figure 5:**
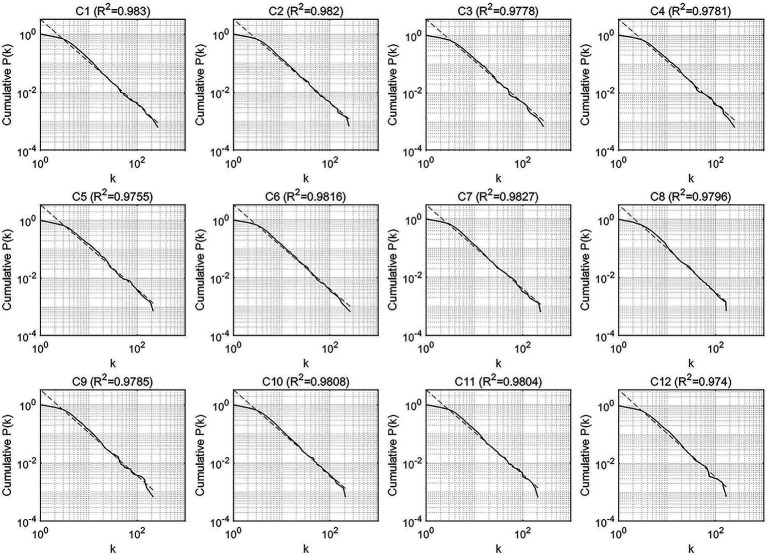
The cumulative degree distributions of the syntactic dependency networks of SI outputs.

To investigate the global characteristics of the output networks, the scale-free and small-world properties, we first observed the cumulative degree distributions of the two types of syntactic dependency networks, respectively, for the evaluation of the scale-free feature. Figures 4, 5 display the cumulative degree distributions (in log–log scales) of SI and CI output networks.

Obviously, all the cumulative degree distributions of CI and SI networks followed the power law with all the determination coefficients R^2^ above 0.9, indicating that the degrees of CI and SI fit the power-law distribution well and all the networks possess the scale-free property.

With regard to the small-world property, the values of average path length and clustering coefficient of the syntactic dependency networks and their corresponding random networks yielded expected results. As is introduced in the previous section, the small-world structure of a complex network is defined by two key parameters: the average path length *L* and the clustering coefficient *C*. To determine this property precisely, *C_rand_* and *L_rand_* of all networks were calculated first, and then the values of S were obtained. Statistical results of key parameters measuring small-worldness of input and output networks are displayed in Tables 3 and 4, respectively. According to the above-mentioned measure of small-worldness (Humphries and Gurney, 2008), since the values of S of all these networks all satisfied S≫1, all the networks possess the small-world property.

**Table 3:** Results of key parameters measuring network small-worldness (networks of input texts).

Networks of input texts	C	L	*C_rand_*	*L_rand_*	S
CI-1	0.041	3.671	0.004	4.418	11.250
CI-2	0.045	3.681	0.005	4.417	11.747
CI-3	0.048	3.601	0.005	4.236	10.419
CI-4	0.042	3.638	0.005	4.353	10.088
CI-5	0.050	3.610	0.005	4.227	11.227
CI-6	0.042	3.687	0.003	4.402	14.701
CI-7	0.045	3.701	0.004	4.540	14.915
CI-8	0.046	3.761	0.005	4.482	10.926
CI-9	0.042	3.663	0.003	4.474	15.858
CI-10	0.054	3.525	0.004	4.205	14.819
CI-11	0.049	3.754	0.005	4.385	12.642
CI-12	0.045	3.789	0.005	4.499	11.211
SI-1	0.064	3.868	0.005	4.316	14.550
SI-2	0.058	3.862	0.005	4.425	13.007
SI-3	0.028	3.596	0.003	4.526	10.125
SI-4	0.030	3.637	0.003	4.490	10.839
SI-5	0.053	3.867	0.003	4.498	21.173
SI-6	0.049	4.000	0.004	4.676	12.905
SI-7	0.062	3.813	0.004	4.412	19.713
SI-8	0.056	3.864	0.003	4.430	20.063
SI-9	0.049	3.946	0.004	4.527	12.677
SI-10	0.051	3.916	0.004	4.538	15.444
SI-11	0.060	3.747	0.006	4.258	11.599
SI-12	0.049	3.920	0.003	4.654	17.895

**Table 4:** Results of key parameters measuring network small-worldness (networks of output texts).

Networks of output texts	C	L	*C_rand_*	*L_rand_*	S
CI-1	0.032	3.496	0.004	4.212	9.750
CI-2	0.034	3.425	0.004	4.084	9.887
CI-3	0.037	3.388	0.004	4.010	9.798
CI-4	0.032	3.484	0.004	4.105	9.209
CI-5	0.037	3.495	0.005	4.114	9.259
CI-6	0.033	3.476	0.005	4.151	8.489
CI-7	0.030	3.527	0.004	4.224	8.549
CI-8	0.030	3.684	0.004	4.361	8.402
CI-9	0.030	3.561	0.005	4.198	7.097
CI-10	0.032	3.536	0.004	4.185	10.363
CI-11	0.037	3.544	0.005	4.161	9.114
CI-12	0.032	3.621	0.004	4.213	8.261
SI-1	0.033	3.512	0.004	4.251	9.343
SI-2	0.030	3.576	0.003	4.376	11.403
SI-3	0.028	3.596	0.004	4.425	8.435
SI-4	0.030	3.637	0.005	4.410	7.258
SI-5	0.027	3.582	0.004	4.392	8.350
SI-6	0.031	3.583	0.003	4.373	11.438
SI-7	0.033	3.534	0.004	4.376	9.430
SI-8	0.033	3.519	0.004	4.311	9.996
SI-9	0.029	3.586	0.003	4.353	12.002
SI-10	0.034	3.504	0.003	4.299	13.112
SI-11	0.034	3.542	0.004	4.303	11.701
SI-12	0.028	3.674	0.003	4.402	12.469

### Distinctions in Main Parameters of CI and SI Syntactic Dependency Networks

The values of some common-used parameters of SI and CI dependency networks of inputs and outputs are presented in Appendix I in Supplementary Material. To investigate the discrepancy of CI and SI output network properties by excluding the interference from the input values, a series of one-way ANCOVAs were conducted. As is shown in Table 5, significant differences were observed in the number of vertices (N), *F*(1,21)=23.675, *p*<0.001, partial η^2^=0.53, and the estimated marginal means showed that SI networks had more vertices than CI networks (M=1493.47, SD=19.725 for CI, M=1641.53, SD=19.725 for SI). Significant differences also existed in average degree [<k>; *F*(1,21)=16.511, *p*=0.001, partial η^2^=0.44], clustering coefficient [C; *F*(1,21)=8.019, *p*=0.01, partial η^2^=0.276], density [ρ; *F*(1,21)=48.166, *p*<0.001, partial η^2^=0.696], betweenness centrality [*F*(1,21)=51.636, *p*<0.001, partial η^2^=0.711], and degree centrality [*F*(1,21)=22.916, *p*<0.001, partial η^2^=0.522]. Comparing the estimated marginal means, the results showed that, for parameter <k>, the average degree of CI output networks was significantly larger than that of SI networks (M=6.318, SD=0.06 for CI, M=5.971, SD=0.06 for SI); for parameter C, CI output networks had a larger clustering coefficient than SI (M=0.33, SD=0.01 for CI, M=0.30, SD=0.01 for SI); for parameter ρ, the networks of CI yielded a larger density than SI (M=0.0042, SD=0.00 for CI, M=0.0037, SD=0.00 for SI); and for betweenness centrality and degree centrality, SI was significantly larger than CI (betweenness centrality: M=0.196, SD=0.009 for CI, M=0.297, SD=0.009 for SI; degree centrality: M=0.13, SD=0.006 for SI, M=0.185, SD=0.006 for SI). However, no significant difference of the shortest path length (L; *p*=0.38) and diameter (D; *p*=0.479) was observed between SI and CI.

**Table 5:** Results of ANCOVAs on main parameters of CI and SI output networks.

Measures	Networks	N	Mean	SD	Adjusted mean	*F*	Partial η2
N	CI outputs	12	1475.17	62.98	1493.47	23.675^**^	0.53
	SI outputs	12	1659.83	69.38	1641.53		
	CI outputs	12	6.324	0.283	6.318	16.511^**^	0.44
	SI outputs	12	5.964	0.138	5.971		
C	CI outputs	12	0.0329	0.0027	0.033	8.019^*^	0.276
	SI outputs	12	0.0308	0.0025	0.03		
L	CI outputs	12	3.52	0.08	3.529	0.804	
	SI outputs	12	3.57	0.051	3.561		
D	CI outputs	12	8.417	0.669	8.391	0.52	
	SI outputs	12	8.583	0.793	8.609		
Density	CI outputs	12	0.00429	0.00022	0.0042	48.166^**^	0.696
	SI outputs	12	0.0036	0.00017	0.0037		
BC	CI outputs	12	0.202	0.024	0.196	51.636^**^	0.711
	SI outputs	12	0.29	0.027	0.297		
DC	CI outputs	12	0.149	0.021	0.13	22.916^**^	0.522
	SI outputs	12	0.165	0.014	0.185		

BC, betweenness centrality; DC, degree centrality.

* *p*<0.05;

** *p*<0.01.

### Characteristics of Central Vertices in SI and CI

The degree centrality and betweenness centrality of SI and CI output networks demonstrated significantly different results, suggesting that distinctions exist in the prominence of vertices in the SI and CI networks from a global perspective. However, the centrality of each vertex, which is a proxy for the combinatorial capacity of a certain linguistic unit behaving as a hub, is also worthy of examination. Vertices with higher centrality behave as the powerful hubs of a network, representing more importance and a stronger combinatorial capacity. It is well documented that, in a syntactic dependency network, the hubs have the tendency to be function words (e.g., articles, conjunctions, and prepositions; Ferrer-i-Cancho and Solé, 2001; Solé et al., 2010), which play an important role in identifying grammatical relationships and the structure of a sentence (Jin and Liu, 2016). In the context of interpreting, the comparison of function words used in the interpreted speech among the highly connective linguistic units between SI and CI may reflect different processing mechanisms and cognitive demand in diverse interpreting modes.

Hence, to probe into the characteristics of central vertices in the networks of SI and CI, we investigated the percentages of function words in the top 20 values of betweenness centrality and degree centrality. The results are shown in Table 6.

**Table 6:** The percentages of function words in the top 20 values of betweenness centrality and degree centrality.

Network number	SI outputs (BC; %)	CI outputs (BC; %)	SI outputs (DC; %)	CI outputs (DC; %)
1	45	55	60	70
2	40	60	50	75
3	60	50	60	65
4	50	55	65	65
5	45	50	60	60
6	45	55	55	70
7	40	60	50	60
8	40	55	60	70
9	45	50	60	70
10	40	55	50	70
11	40	60	55	70
12	50	55	60	65

BC, betweenness centrality; DC, degree centrality.

**Figure 6:**
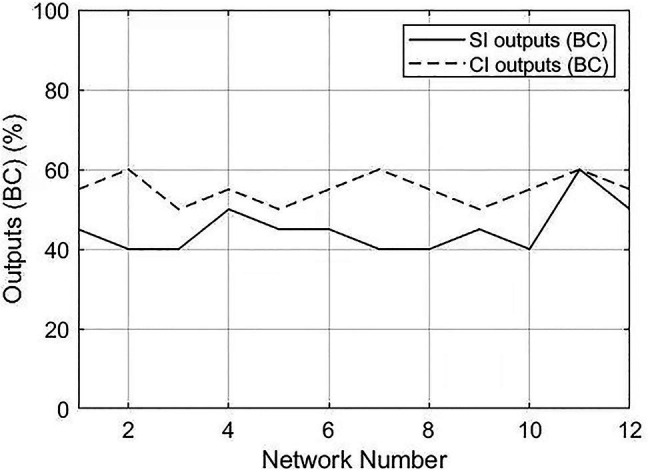
Distributions of percentages of function words in the top 20 values between SI and CI outputs. BC, betweenness centrality.

**Figure 7:**
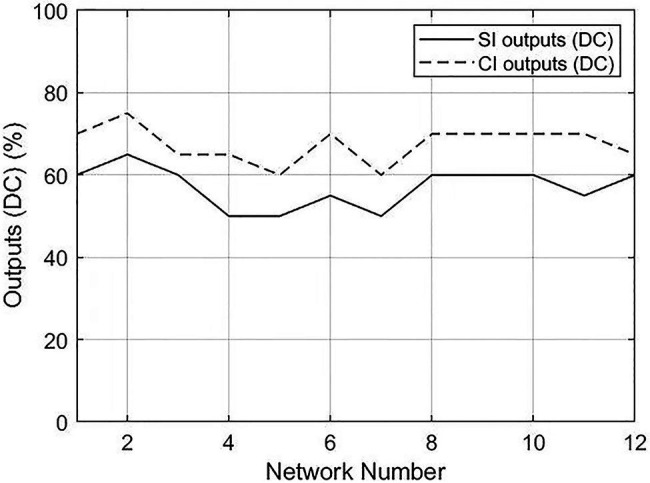
Distributions of percentages of function words in the top 20 values between SI and CI outputs. DC, degree centrality.

The Mann–Whitney U test showed that significant differences existed between SI outputs and CI outputs in both betweenness centrality (*p*=0.001) and degree centrality (*p*<0.001). The difference of distribution between CI and SI output texts is illustrated in Figures 6, 7. The percentages of CI outputs were significantly higher than SI outputs for both betweenness centrality and degree centrality.

## Discussion

The current research examined the properties of syntactic dependency networks of SI and CI outputs. To our knowledge, this is the very first effort in quantifying interpreting types by adopting the complex networks approach from a macroscopic perspective, complementing to previous findings pertaining to the features of individual linguistic units.

Our results showed that, firstly, all the networks of SI and CI exhibited the scale-free and small-world properties. The syntactic networks of CI showed higher degrees, clustering coefficients, and density with a larger number of vertices, suggesting that CI has a higher connectivity between vertices and a higher transitivity of information than SI. Second, the parameter of the network centrality can differentiate CI and SI syntactic dependency networks. CI networks demonstrate a more important role of function words among the central vertices than SI networks.

### Small-World and Scale-Free Properties of SI and CI Networks

Judging from the observed data, all the networks of SI and CI displayed the small-world properties. According to measurement of small-world structure introduced in the previous section, the small-world structure is determined by clustering coefficient and the shortest path length. The clustering coefficient indicates that two neighbors of a given vertices are also connected to each other. As contrast to the corresponding random networks, real networks with the small-world structures have much greater clustering coefficients (Watts and Strogatz, 1998). Our results indicate that the neighbors of a given vertex are highly interconnected in both SI and CI networks, which promotes the efficiency of transmitting the message from the input language and eases the cognitive burden imposed in the interpreting processes.

Similarly, a network with the small-world structure has a shorter average path length which denotes higher global efficiency of information transmission. In the process of the interpreting production, interpreters have to retrieve the expressions and information stored in the long-term memory as soon as possible as well as comprehending the source message simultaneously. As proposed by Collins and Loftus (1975), the fundamental mechanism of information retrieval in the human mind is spreading activation, and the resources allocated to the activation can be inevitably consumed during the course of spreading. The small-world topology observed in the SI and CI syntactic networks reveals that each pair of vertices can be linked by a small path length, thus minimizing the loss of activation energy and maximizing the success of the information retrieval during the process of interpreting. Moreover, given the associations between the shortest path length and dependency distance (Liu, 2008b), Our findings are also in line with the tendency of minimization of dependency distance in natural language (Liu, 2008b; Futrell et al., 2015; Liu et al., 2017) and adhere to the principle of Least Effort (Zipf, 1949).

The scale-free structure of SI and CI syntactic networks denotes the heterogeneity of connectivity of vertices. Our results showed the power-law-like distribution of degree, suggesting that only a small number of linguistic units have extremely great combinatorial capacity while most vertices have rather low degrees. Hence, the scale-free networks of SI and CI possess the properties of “extreme resilience to random deletion of vertices coupled with extreme sensitivity to the targeted deletion of the most connected vertices” (Albert et al., 2000; Baronchelli et al., 2013).

Furthermore, the non-trivial properties of networks of CI and SI are in line with the findings by Liu (2008a), in which Chinese syntactic dependency networks are observed to exhibit similar patterns. These results converge to reinforce the universality of such network patterns in human languages (Ferrer-i-Cancho et al., 2004; Ferrer-i-Cancho, 2005).

### Distinctions in Main Parameters of CI and SI Syntactic Dependency Networks

The disparity of the properties in SI and CI networks can be explicated by cognitive mechanisms during SI and CI and thus have cognitive implications.

The most important findings point to the distinctions between average degree and clustering coefficient of CI and SI. The average degree, on the one hand, denotes the efficiency of a word type being used in the texts. Our results showed that, with a similar size of word tokens, the average degrees of CI networks were significantly larger than SI, suggesting that the same word types are more repeatedly and frequently used in CI networks. This is in line with our result of a lower degree of lexical diversity in CI networks, which provides support for the heavier cognitive load demanded in CI. As postulated by Cowan (1999), working memory is a complex construct which contains activated elements of long-term memory, and the focus of attention is basically capacity limited. In SI, the input information is comprehended in segments, and only the most local content is stored in working memory. The cognitive load for accommodating and processing the chunks of information can instantly be relieved once it is interpreted (Liang et al., 2017). However, CI interpreters need to hold more chunks of information in the focus of attention, and the cognitive load may keep accelerating and accumulating. Additionally, due to limited time of note-taking, CI interpreters can take down only part of information. Thus, the cognitive burden from memorizing long chunks of information and insufficient note-taking information may result in heavier cognitive load in the reformulation phase in CI than in SI. To reduce the processing burden, CI interpreters have an inherent tendency toward using less-varied words.

On the other hand, in the context of a syntactic dependency network, the degree of a given vertex implies the combinatorial capacity of a word to form syntactic dependency relations. The average degree of a whole network reflects the connectivity between linguistic units in the syntactic sub-system (Cong and Liu, 2014a). The higher average degree of CI networks demonstrates the better connectivity and communicating efficiency in CI than SI. Such an efficient organizational sub-system of CI may be due to the accumulation of long chunks of information from the source language before articulating the interpreted speech. A higher average degree of CI networks thus greatly facilitates the understanding of the original message as a whole and enables CI interpreters to reformulate the intended meaning in a comprehensive way. By contrast, the process of SI requires the simultaneity of listening and production. The lag between the hearing of the source speech segment and its corresponding reformulation (the Ear-Voice Span) is much shorter (Gile, 2009). To avoid the potential overloading of memory, interpreters sometimes even shorten the lag to reduce the requirements of short-term memory, which may deprive SI interpreters of adequate understanding and increase the risks of misunderstanding and infelicities such as the incompleteness of the sentence structure and incoherence of the target information (Gile, 2009). In such cases, a smaller volume of information stored in the short-term memory for comprehension may result in lower degree of connectivity in SI output networks, while the overall grasp of intended meaning and the integration of more information segments in CI contributes to the enhancement of connectivity in CI output networks.

As a quantitative measure of transitivity, the clustering coefficient offers another perspective for the understanding of cognitive complexity (Watts and Strogatz, 1998; Baronchelli et al., 2013). The higher clustering coefficient values in CI networks indicate a better transitivity of information in CI than SI, which is consistent with the result of density that the probability of any pair of CI linguistic unit to be involved in a relation is larger than SI. Given the high memory load during CI reformulation phase which has been explicated above, these findings suggest a natural requirement for CI interpreters to lessen cognitive burden by generating less cognitively costly output. The evidence of the association between cognitive costs and clustering coefficient has been presented in several studies (Vitevitch, 2006, 2008; Goldstein and Vitevitch, 2014), among which it has been demonstrated that words with a high clustering coefficient were responded more quickly than words with a low clustering coefficient (Vitevitch, 2006). Hence, in this case, to save more time and energy for the reformulation of the unfolding sentences, CI interpreters are inclined to retrieve words that have more syntactic relations with each other.

The average clustering coefficient of a network is a parameter of local communitization, whereas the average path length is a proxy for macro-scale communitization in a network (Borodkin et al., 2016). The higher clustering in CI networks helps ease the cognitive burden and facilitates the local processing of information segments in CI. It is noted that the average path lengths and diameter of CI and SI networks were similar, suggesting that the two types of networks have no significant difference in terms of the macro-scale communitization. Although the disparity of local processing exists between SI and CI due to various cognitive demands in the processes of interpreting, the global integration of information in the process of SI and CI remains the same in a broader sense.

Besides the average degree and clustering coefficient, several other parameters that distinguish SI and CI are worthy to be discussed. Since the vertices in a syntactic dependency network represent word types, our finding that the number of SI vertices was significantly higher than CI indicates higher degree of vocabulary richness and lexical complexity in SI. This result, on the one hand, might stem from the fact that CI interpreters tend to build a mental map which focuses on interconnections and general architecture of the intended meaning, without much attention to lexical reformulation. This is consistent with previous findings that CI output texts yields more simplified outputs than SI (Lv and Liang, 2019). On the other hand, the differences in lexical complexity might arise from cognitive load. According to the Effort Model (Gile, 2009), interpreting is conceived as comprising functional “efforts” which compete with each other in terms of processing capacity, and thus the increments of one effort will result in the decrements in another. Given that CI interpreters have to maintain a large volume of information segments from the source language, the burden on the memory is constantly accumulating to saturation in the reformulation phase. Moreover, due to the long time lag between listening and production, reformulation during CI is more self-paced and independent from the source structure (Gile, 2005). In this case, CI interpreters may tend to use less sophisticated and more repetitive vocabulary to reduce cognitive efforts (Liang et al., 2017; Lv and Liang, 2019). On the contrary, SI interpreters generally maintain the syntactic structure of source speech due to the simultaneity of perception and production in SI (Bacigalupe, 2010), and the cognitive load for the storage of previous information segments can soon be alleviated (Liang et al., 2017). Thus, lexical access, even of complex words, might be less challenging for the mind than syntactic reformulation in SI.

With regard to the network property of centralization, degree centralization and betweenness centralization can differentiate SI and CI syntactic dependency networks. Degree centralization reflects the degree of variation among the vertices, and betweenness centralization indicates the tendency of a network to exhibit a star-like topology. Here in the present study, a larger degree of variation was observed in SI compared with CI. Among all the vertices in a network, linguistic units with relatively greater connectivity are more important, and these powerful vertices are regarded as hubs (Cong and Liu, 2014a). Convergent evidence suggests that hubs tend to be function (grammatical) words (e.g., Ferrer-i-Cancho and Solé, 2001; Solé et al., 2010; Cong and Liu, 2014a; Jin and Liu, 2016). Given the significance of function words in identifying grammatical relationships as well as its potential to reflect different processing mechanisms and cognitive complexity in diverse interpreting modes, the following section will discuss the proportion of function words in the hubs of SI and CI networks.

### Characteristics of Central Vertices in SI and CI

In exploring the characteristics of central vertices, our result showed that the percentages of function words in the hubs of CI networks were higher than SI networks. This indicates a better use and higher importance of function words in the processes of CI, conforming to previous findings that function words are conceived to be more densely distributed in CI than SI output (Liang et al., 2019). The distinction of the proportion of function words among the central vertices in CI and SI networks may be explicated by different processing mechanisms and cognitive complexity in CI and SI.

For SI interpreters, the interpretation begins immediately after the articulation of the original speech, with several units of information stored in the working memory. According to Cowan (1995), the number of units that can be held in the “focus of attention” in working memory is said to be 3 to 4 chunks. Constrained by the limited cognitive resources, the outputs of SI generally follow the structure of the original chunks without much altering the order of the input text segments (Bacigalupe, 2010; Liang et al., 2017, 2019). To minimize the chunks held in the working memory, the SI production needs to be generated in an efficient way, which renders interpreters to give priority to the content words with specific meanings other than the function words which has limited meaning but occupy the cognitive resources in the process of word selections. In this way, not only can the meaning of the source speech achieve the maximum retention, but also the efficient processing of the current information segments reduces the number of items to be held in the working memory and avoids any delay of the processing of subsequent chunks (Mizuno, 2017). Moreover, as suggested by Hatim and Mason (2002), because the reception and production of the text occur almost simultaneously, SI interpreters receive the input in piecemeal, and thus they generally make tentative anticipations of the context and structure. Since these various hypotheses have to be confirmed or disapproved by the incoming detailed information, SI interpreters “rely more heavily on the emerging texture in order to make and maintain sense. In other words, the rich variety of detailed information must be “relied upon the most tangible point of reference” (Hatim and Mason, 2002). The content words, which are variable and rich in meaning, thus outweigh the function words for SI interpreters when handling the production.

By contrast, given the distinctive feature of CI that the utterance of the production has a long time lag after the source speech, interpreters start interpreting when a large proportion of the input information is fully processed, and hence, they can have a holistic comprehension of the original meaning. With longer segments stored in the working memory, CI interpreters tend to discard the linguistic form of the source language and restructure the sentence instead of interpreting in the word-for-word consistency to relieve the pressure (Ouyang, 2018). That means, the extra load on memory makes it hard to retain the detailed information of the texture and context from the input text. To achieve smooth and successful production, CI interpreters generally utilize the manifestation of the detailed information as the means to arrange the structure in a comprehensive manner (Hatim and Mason, 2002). Such a way of relying on the structure in CI outputs indicates that the use of function words is of vital necessity, since function words define and signal sentence structure. As claimed by Gervain et al. (2013), function words act as entry points “with respect to which the structural roles and sequential positions of other constituents can be encoded and remembered.” Consequently, the role of function words in CI is demonstrated to be more significant than in SI. CI interpreters may resort to the function words more frequently than in the SI tasks to produce smooth and coherent outputs with the added pressure on the memory.

### Connections Between Complex Network and Cognition

The networks we constructed provide important insights into a deeper understanding of cognition by capturing the interplay between the structure of the underlying networks and cognitive processes operating in interpreting. The small-world structure of SI and CI outputs networks has close associations with the domain of cognition. As claimed by Siew et al. (2019), the small-world structure “may emerge from systematic growth processes that may adapt to environmental constraints to give rise to a beneficial structure.” Considering the context of interpreting which represents a special case of language use under the extreme cognitive load, such a structure may mirror the trade-off effect between the path length among the words and the cost of creating connections between the words. A similar process has been found in the brain networks, and these findings support the view that the small-world structure may offer a quantitative means to optimize the organizational structure (Bullmore and Sporns, 2012). It also provides a vital clue into the representation of the structure of cognitive systems which can maximize the efficiency of interpreting processes under the cognitive constraints and minimize cognitive load of interpreting in the language system. In short, the construction of interpreting networks demonstrates how different types of interpreting tasks mediate the global structural organization of the interpreting outputs to achieve cognitive load minimization. This echoes previous research quantifying interpreting types by language sequence (Liang et al., 2019).

Our results also indicate that the networks of interpreting outputs are inherently dynamic due to different cognitive constraints in various modes of interpreting processes. The properties and representations of interpreting networks vary among different manipulations in the interpreting processes. We posit that the disparate properties between CI and SI networks reflect different cognitive processes operating in interpreting. As is shown in our findings, CI output networks display a better connectivity, more efficient transitivity and higher importance of function words than SI. These results show the convergent evidence of a more efficient organizational sub-system of CI, which may be partly associated with a cognitive load accumulation in CI and a cognitive load relief process in SI. Therefore, our study demonstrates the role of complex network in re-conceptualizing the cognitive processes of complex interpreting tasks as dynamic processes. This novel approach offers an avenue to explore the processing mechanisms of interpreting that lead to cognitive insights.

Furthermore, the measure of complex networks in the current research, together with such a quantitative approach as the calculation of dependency distance (Liang et al., 2017) and the quantification of lexical features in the output interpreting texts (Lv and Liang, 2019) which are adopted in the prior studies, serves as a means to calculate the cognitive processing underlying different types of interpreting tasks. Hence, information processing is an important concept in understanding how interpreting processes operate, and it points toward computing as a fundamental instrumental approach for modeling and exploring cognitive complexity (Cioffi-Revilla, 2014).

In synthesizing the above analyses, our study provides valuable evidence from the domain of interpreting to support the view that the network of interconnected vertices is closely connected with the study of cognition. The complex network offers a unifying framework to investigate different system under the same conceptual lens and facilitates our understanding of cognitive processes (Barabási, 2011; Baronchelli et al., 2013).

## Conclusion

In the present study, we examine the properties of syntactic dependency networks of SI and CI outputs. We find that both SI and CI networks exhibit the scale-free and small-world structures, corroborating the universality of such network patterns in human language. Most of the network parameters can discriminate SI and CI networks, and CI networks demonstrate higher degrees, clustering coefficients and density with a larger number of vertices, suggesting a better connectivity, transitivity and a lower degree of vocabulary richness in CI outputs. These may be ascribed to the constant accumulation of memory burden as well as the long chunks of information in the process of CI. In terms of the characteristics of central vertices, our results also reveal the higher importance of function words in the processes of CI, which can be explicated by different underlying mechanisms and cognitive complexity in CI and SI.

Our study offers a valuable integrative framework for the understanding of processing mechanisms during SI and CI, shedding light on interpreting training. Specific strategies can be given to interpreters in reference to distinctive cognitive features and coping mechanisms of a particular interpreting type. The findings also offer insights into the artificial intelligence that cognitive factors can be integrated into the development of the machine translation to approach human cognition.

## Data Availability Statement

The original contributions presented in the study are included in the article/**Supplementary Material**, further inquiries can be directed to the corresponding authors.

## Author Contributions

YL, DX, and JL conceived and designed the experiments, performed the experiments and data analyses, and collected the data. All authors contributed to the interpretation of results, the writing of the manuscript, and approved the final version of the manuscript for submission.

## Funding

This work was partly supported by the National Social Science Foundation of China (Grant No. 17BYY068), the Fundamental Research Funds for the Central Universities of China (Programme of Big Data PLUS Language Universals and Cognition, Zhejiang University), and the Zhejiang Provincial Teaching Reform Project (Grant No. JG20180014).

## Conflict of Interest

The authors declare that the research was conducted in the absence of any commercial or financial relationships that could be construed as a potential conflict of interest.

## Publisher’s Note

All claims expressed in this article are solely those of the authors and do not necessarily represent those of their affiliated organizations, or those of the publisher, the editors and the reviewers. Any product that may be evaluated in this article, or claim that may be made by its manufacturer, is not guaranteed or endorsed by the publisher.

## Footnotes

^1^Createpajek can be downloaded from http://vlado.fmf.uni-lj.si/pub/networks/pajek/howto/excel2pajek.htm

^2^Pajek is used for analysis and visualization of large networks. It can be downloaded from http://mrvar.fdv.uni-lj.si/pajek/default.htm
