# Supplementary material for: Differentiating Interpreting Types: Connecting Complex Networks to Cognitive Complexity
Source: Front Psychol. 2021 Sep 17;12:590399. doi: 10.3389/fpsyg.2021.590399 (PMC8484889; doi:10.3389/fpsyg.2021.590399)
Supplement: Supplementary file 1 [file Table_1.DOCX]

| SI-12 | SI-11 | SI-10 | SI-9 | SI-8 | SI-7 | SI-6 | SI-5 | SI-4 | SI-3 | SI-2 | SI-1 | CI-12 | CI-11 | CI-10 | CI-9 | CI-8 | CI-7 | CI-6 | CI-5 | CI-4 | CI-3 | CI-2 | CI-1 | Input  Texts | Table 1 *Summary of main parameters of input SI and CI syntactic dependency networks* |
| --- | --- | --- | --- | --- | --- | --- | --- | --- | --- | --- | --- | --- | --- | --- | --- | --- | --- | --- | --- | --- | --- | --- | --- | --- | --- |
| 1398 | 1372 | 1487 | 1562 | 1374 | 1386 | 1479 | 1431 | 1561 | 1420 | 1304 | 1309 | 1396 | 1309 | 1408 | 1377 | 1218 | 1405 | 1332 | 1217 | 1404 | 1252 | 1192 | 1228 | N |  |
| 3531 | 4070 | 3985 | 4241 | 3769 | 3859 | 3762 | 3876 | 4317 | 3762 | 3535 | 3756 | 3767 | 3618 | 4291 | 3717 | 3176 | 3722 | 3688 | 3539 | 4025 | 3679 | 3187 | 3307 | E |  |
| 5001 | 5230 | 5025 | 5150 | 4789 | 4960 | 4600 | 5010 | 5248 | 4577 | 4712 | 4839 | 4631 | 4553 | 5493 | 4635 | 4157 | 4668 | 4607 | 4488 | 5265 | 4766 | 4263 | 4166 | Tokens |  |
| 5.05150215 | 5.93294461 | 5.3597848 | 5.43021767 | 5.48617176 | 5.56854257 | 5.0872211 | 5.41719078 | 5.84894614 | 5.81743227 | 5.42177914 | 5.73873186 | 5.39684814 | 5.52788388 | 6.09517045 | 5.39869281 | 5.21510673 | 5.29822064 | 5.53753754 | 5.81594084 | 5.73361823 | 5.87699681 | 5.34731544 | 5.38599349 | <k> |  |
| 0.04860247 | 0.05973054 | 0.05084489 | 0.04857146 | 0.05619273 | 0.06200791 | 0.04871464 | 0.0525541 | 0.02995249 | 0.02800439 | 0.0583821 | 0.06422402 | 0.04498548 | 0.04884653 | 0.05357016 | 0.04247929 | 0.04608975 | 0.04451171 | 0.04197025 | 0.05039616 | 0.04199552 | 0.04846424 | 0.04528302 | 0.04121965 | C |  |
| 3.91969 | 3.74707 | 3.91642 | 3.94576 | 3.86357 | 3.81259 | 3.99975 | 3.86731 | 3.63749 | 3.59575 | 3.86235 | 3.86782 | 3.78939 | 3.75398 | 3.52526 | 3.66311 | 3.76115 | 3.70133 | 3.68679 | 3.60953 | 3.63782 | 3.60147 | 3.6805 | 3.67116 | L |  |
| 10 | 9 | 10 | 11 | 10 | 9 | 11 | 9 | 9 | 8 | 9 | 9 | 10 | 9 | 8 | 9 | 10 | 9 | 9 | 9 | 9 | 10 | 9 | 10 | D |  |
| 0.00361596 | 0.00432746 | 0.00360685 | 0.00347868 | 0.00399576 | 0.00402061 | 0.00344196 | 0.00378825 | 0.00354556 | 0.00373403 | 0.004161 | 0.00438741 | 0.00386871 | 0.00422621 | 0.00433203 | 0.00392347 | 0.00428522 | 0.00377366 | 0.00416043 | 0.00478285 | 0.00408668 | 0.00469784 | 0.00448977 | 0.00438956 | Density |  |
| 0.301396 | 0.2111092 | 0.2298918 | 0.1909557 | 0.2099044 | 0.2038894 | 0.2174662 | 0.2262097 | 0.258977 | 0.2650146 | 0.2418231 | 0.2587493 | 0.2253504 | 0.247533 | 0.2825224 | 0.2942685 | 0.2711301 | 0.297094 | 0.3465698 | 0.320668 | 0.3233575 | 0.3236042 | 0.3237479 | 0.3815568 | Betweennes  Centrality |  |
| 0.12756152 | 0.11400065 | 0.11026959 | 0.09465661 | 0.11853658 | 0.12106383 | 0.10427685 | 0.11455368 | 0.1222521 | 0.11199806 | 0.11420296 | 0.12960266 | 0.12965037 | 0.14201524 | 0.1707506 | 0.1561871 | 0.14303264 | 0.15955888 | 0.17265779 | 0.18054676 | 0.18291657 | 0.18744812 | 0.16959832 | 0.18825608 | Degree  Centrality |  |

| SI-12 | SI-11 | SI-10 | SI-9 | SI-8 | SI-7 | SI-6 | SI-5 | SI-4 | SI-3 | SI-2 | SI-1 | CI-12 | CI-11 | CI-10 | CI-9 | CI-8 | CI-7 | CI-6 | CI-5 | CI-4 | CI-3 | CI-2 | CI-1 | Output Texts | Table 2 *Summary of main parameters of output SI and CI syntactic dependency networks* |
| --- | --- | --- | --- | --- | --- | --- | --- | --- | --- | --- | --- | --- | --- | --- | --- | --- | --- | --- | --- | --- | --- | --- | --- | --- | --- |
| 1693 | 1665 | 1641 | 1821 | 1624 | 1567 | 1593 | 1695 | 1708 | 1698 | 1621 | 1592 | 1395 | 1508 | 1464 | 1459 | 1409 | 1537 | 1482 | 1396 | 1553 | 1450 | 1455 | 1594 | N |  |
| 4936 | 5091 | 5012 | 5557 | 4913 | 4593 | 4677 | 4990 | 4995 | 4939 | 4766 | 4932 | 4224 | 4795 | 4559 | 4519 | 4032 | 4774 | 4722 | 4471 | 5153 | 4944 | 4770 | 5042 | E |  |
| 6208 | 6834 | 6615 | 7117 | 6429 | 5905 | 6010 | 6371 | 6491 | 6323 | 6214 | 6632 | 5248 | 6957 | 5853 | 5793 | 5250 | 6307 | 6310 | 6203 | 7075 | 6977 | 6519 | 6719 | Tokens |  |
| 5.83106911 | 6.11531532 | 6.10847044 | 6.10323998 | 6.05049261 | 5.86215699 | 5.87193974 | 5.8879056 | 5.84894614 | 5.81743227 | 5.88032079 | 6.1959799 | 6.05591398 | 6.35941645 | 6.22814208 | 6.19465387 | 5.72320795 | 6.2121015 | 6.37246964 | 6.40544413 | 6.63618802 | 6.81931034 | 6.55670103 | 6.32768362 | <k> |  |
| 0.02809732 | 0.03431249 | 0.03362791 | 0.02875208 | 0.03317334 | 0.03252092 | 0.03099683 | 0.02710491 | 0.02995249 | 0.02800439 | 0.03015407 | 0.03272587 | 0.03151293 | 0.03672549 | 0.03242058 | 0.0295731 | 0.03004867 | 0.0304792 | 0.03267708 | 0.03683524 | 0.03183617 | 0.03708768 | 0.03364277 | 0.03156867 | C |  |
| 3.67427 | 3.54169 | 3.5036 | 3.58636 | 3.51866 | 3.53442 | 3.58283 | 3.58208 | 3.63749 | 3.59575 | 3.57566 | 3.51243 | 3.62073 | 3.54362 | 3.53643 | 3.56103 | 3.68443 | 3.52661 | 3.47633 | 3.49465 | 3.48372 | 3.38842 | 3.42453 | 3.49594 | L |  |
| 10 | 8 | 8 | 8 | 8 | 10 | 8 | 9 | 9 | 8 | 8 | 9 | 8 | 9 | 8 | 9 | 9 | 8 | 9 | 9 | 9 | 7 | 8 | 8 | D |  |
| 0.00344626 | 0.00367507 | 0.00372468 | 0.00335343 | 0.00372797 | 0.0037434 | 0.0036884 | 0.00347574 | 0.00342645 | 0.00342807 | 0.00362983 | 0.00389439 | 0.00434427 | 0.00421992 | 0.0042571 | 0.00424873 | 0.00406478 | 0.00404434 | 0.00430282 | 0.00459172 | 0.00427589 | 0.00470622 | 0.00450942 | 0.00397468 | Density |  |
| 0.13146081 | 0.17923253 | 0.16354782 | 0.15890901 | 0.1727016 | 0.17400104 | 0.16045777 | 0.16082264 | 0.16137881 | 0.15821969 | 0.18054355 | 0.18363989 | 0.11562063 | 0.13331588 | 0.13810518 | 0.14409694 | 0.11399476 | 0.14914453 | 0.1721618 | 0.15046256 | 0.15571846 | 0.18257114 | 0.1655944 | 0.16635674 | Betweenness Centrality |  |
| 0.23412168 | 0.31504863 | 0.26017647 | 0.28458633 | 0.30205168 | 0.29545587 | 0.27137799 | 0.28715062 | 0.30113903 | 0.28640663 | 0.33438699 | 0.31100948 | 0.16422604 | 0.17157234 | 0.18949208 | 0.18861431 | 0.2072828 | 0.21749336 | 0.24126045 | 0.19491296 | 0.18745415 | 0.22999096 | 0.19802794 | 0.23110291 | Degree Centrality |  |
